# Molecular Design of Difluorinated Polyether Electrolyte for Ultrastable High‐Voltage All‐Solid‐State Lithium Metal Batteries

**DOI:** 10.1002/advs.202508721

**Published:** 2025-08-11

**Authors:** Zhenyao Wei, Yufeng Luo, Yongqiang Yang, Yaxin Tang, Junhua Zhou, Chao Luo, Ruo Wang, Huipeng Zeng, Chaoyang Wang, Xiaoxiong Xu, Yonghong Deng, Zijian Zheng, Jian Chang

**Affiliations:** ^1^ Department of Materials Science and Engineering Guangdong Provincial Key Laboratory of Energy Materials for Electric Power Southern University of Science and Technology Shenzhen 518055 China; ^2^ School of Fashion and Textiles The Hong Kong Polytechnic University Hong Kong 999077 China; ^3^ Department of Applied Biology and Chemical Technology The Hong Kong Polytechnic University Hong Kong 999077 China; ^4^ Research Institute of Materials Science South China University of Technology Guangzhou 510640 China; ^5^ Research Institute for Intelligent Wearable Systems The Hong Kong Polytechnic University Hong Kong 999077 China; ^6^ Research Institute for Smart Energy The Hong Kong Polytechnic University Hong Kong 999077 China; ^7^ Dongguan Key Laboratory of Interdisciplinary Science for Advanced Materials and Large‐Scale Scientific Facilities School of Physical Sciences Great Bay University Dongguan Guangdong 523000 China

**Keywords:** all‐solid‐state lithium metal batteries, difluorinated polyether electrolytes, electron‐withdrawing effect, high‐voltage cathode, solid polymer electrolytes

## Abstract

Solid polymer electrolytes with high interfacial stability are considered among the most promising alternatives for replacing liquid electrolytes in high‐voltage lithium (Li) metal batteries. However, their application faces significant challenges, such as random dendrite deposition, interfacial side reactions, and sluggish ion transport, leading to performance degradation and safety hazards. Herein, an inherently stable difluorinated polyether electrolyte (DPE) is proposed that exhibits superior interfacial stability and ion conductivity, enabling the reliable operation of high‐voltage all‐solid‐state Li metal batteries (ASSLMBs). Due to the synergistic electron‐withdrawing and ion solvation effects of difluorinated functional groups, DPE shows an improved oxidation voltage of 4.9 V and high Li^+^ conductivity of 2.0 × 10^−4^ S cm^−1^. The generated LiF‐rich electrolyte/electrode interphase further improves the stability of DPEs against both Li metal anode and high‐voltage cathode. Consequently, the assembled all‐solid‐state Li||LFP battery retains 73.17% of its capacity after 700 cycles. The high‐voltage all‐solid‐state Li||LiNi_0.6_Co_0.2_Mn_0.2_O_2_ (NCM622) battery remains stable over 300 cycles with a high capacity retention of 76.02%. Moreover, the high‐voltage ASSLMB shows negligible capacity degradation during 3000 bending cycles at a small radius curvature of 4.0 mm. This work provides a feasible strategy for designing antioxidant polymer electrolytes for the stable operation of high‐voltage Li metal batteries.

## Graphical abstract

This work develops an in situ polymerized difluorinated polyether electrolytes (DPEs) for all‐solid‐state lithium metal batteries (ASSLMBs). The DPE exhibits superior interfacial stability and ion conductivity, due to the synergistic electron‐withdrawing and ion solvation effects of difluorinated functional groups. The generated LiF‐rich electrolyte/electrode interphase further enables the ASSLMBs with ultra‐stable cycling performance.

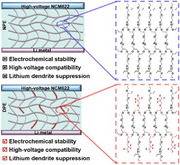

## 1 Introduction

From electric vehicles, and portable devices to wearable devices, various applications are placing increasing demands on rechargeable battery systems with high energy density and reliable safety.^[^ ^1^, ^2^, ^3^ ^]^ Lithium (Li)‐ion batteries using graphite anodes are currently the main power sources on market, but they have reached the theoretical energy density limit. Due to the high theoretical capacity of Li metal anodes (∼3860 mAh g^−1^) and the high operating voltage window of nickel‐rich oxide cathodes (≥4.3 V), high‐voltage Li metal batteries (LMBs) are promising candidates to improve the energy density.^[^ ^4^, ^5^ ^]^ However, serious interface side reactions between electrodes and liquid electrolytes often lead to rapid capacity loss, dendritic short circuits, and flammable hazards during battery operation. Replacing liquid electrolytes by solid electrolytes might potentially eliminate various interface reactions and thermal runaway issues of high‐voltage LMBs.^[^ ^6^, ^7^, ^8^, ^9^ ^]^ Therefore, it is essential to design an inherently stable solid‐state electrolyte that ensure excellent interfacial compatibility, thereby improving the cycling stability and safety of high‐voltage LMBs.

From the perspective of practical application, solid polymer electrolytes (SPEs) are more suitable for fabricating all‐solid‐state LMBs (ASSLMBs) due to their high flexibility, light weight, and easy scalability.^[^ ^10^, ^11^, ^12^ ^]^ Especially, SPEs can form a tight contact at the electrode/electrolyte interface through in‐situ polymerization, effectively reducing interface resistance.^[^ ^13^, ^14^, ^15^, ^16^, ^17^, ^18^ ^]^ In various SPEs, polyether‐based electrolytes show excellent reduction stability for Li metal anodes and have high ion transport capacity due to the unique Li^+^ coordination and dissociation process of ether oxygen (EO) groups.^[^ ^19^, ^20^ ^]^ For example, polyethylene oxide (PEO) as a typical polyether‐based electrolyte has been commercialized in Li‐iron phosphate batteries. However, Li metal anode in polyether‐based electrolytes will still produce dendritic Li deposition during cycling, resulting in continuous capacity loss and thermal runaway risk. In addition, polyether‐based electrolytes are incompatible with nickel‐rich oxide cathodes.^[^ ^21^, ^22^, ^23^ ^]^ On one hand, unstable lone pair electrons on EO groups are prone to causing oxidative decomposition of polymer matrix under high‐voltage conditions; On the other hand, high valence nickel dissolved from the high‐voltage cathode also accelerates this degradation, resulting in uneven electrolyte interface and increased interface resistance.^[^ ^24^, ^25^, ^26^ ^]^ Therefore, ensuring the cycling stability and safety of high‐voltage LMBs through the use of polyether‐based electrolytes remains a huge challenge.

In this regard, various strategies have been proposed to improve the interfacial stability of high‐voltage LMBs emloying polyether‐based electrolytes, including coordination of EO sites with inorganic fillers,^[^ ^27^, ^28^ ^]^ inhibition of catalytic activity by cathodic coatings,^[^ ^22^, ^29^ ^]^ lamination of various polymer electrolytes,^[^ ^30^, ^31^ ^]^ elimination of unstable hydroxyl groups through cross‐linking,^[^ ^32^ ^]^ and molecular fluorination of polymer electrolytes.^[^ ^33^, ^34^ ^]^ Despite significant progress in these strategies, unstable EO bonds still limit the oxidation stability of polyether‐based electrolytes and the cycling stability of high‐voltage LMBs. Among various options, the fluorination strategy has proven to be the most effective strategy to improve the interfacial compatibility of the entire electrolyte at the molecular level.^[^ ^35^ ^]^ Unfortunately, most of the previous reports focused on designing fluorinated polycarbonate electrolytes, but they often have poor interfacial compatibility with Li metal anodes, leading to insufficient cycling performance.^[^ ^36^, ^37^ ^]^ The contact between fluorinated polycarbonate electrolyte and highly active Li metal can cause continuous chemical reduction reactions, resulting in significant interfacial resistance.^[^ ^38^ ^]^ In addition, the introduction of trifluoride functional groups will greatly reduce the ion transport properties of polymer electrolytes by transferring the electron cloud of ion coordination carbonyl groups. Therefore, designing fluorinated polyether electrolytes with superior interfacial stability and ion conductivity is extremely crucial for the stable operation of high‐voltage LMBs under practical conditions.

In this work, we proposed an inherently stable difluorinated polyether electrolyte (DPE) that offers excellent interfacial stability and ion conductivity for the stable operation of high‐voltage ASSLMBs. The DPE is synthesized by one‐step in‐situ cationic ring‐opening copolymerization using polyethylene glycol diglycidyl ether (PEGDE) and glycidyl 2,2,3,3‐tetrafluoropropyl ether (TFE) within a mechanically robust fluorinated mesoporous polymer electrolyte host. Benefiting from the synergistic electron‐withdrawing and ion solvation effects of difluorinated functional groups, the DPE shows a large oxidation potential of 4.9 V (vs Li^+^/Li), high Li^+^ transference number (0.366), and high ionic conductivity (2.0 × 10^−4^ S cm^−1^), which is much better than that of non‐fluorinated polyether electrolyte (NPE). Moreover, the generated LiF‐rich electrolyte/electrode interphase further improves the stability of DPE against both Li metal anode and high‐voltage cathode. The Li||Li symmetric cell with DPE can be stably cycled with an extremely small overpotential of 20 mV at a current density of 0.2 mA cm^−2^ for over 2000 h. In addition, the assembled all‐solid‐state Li||LFP battery with DPE shows long‐term cycling stability with a high capacity retention of 73.17% after 700 cycles. The high‐voltage all‐solid‐state Li||NCM622 battery can be stably operated for 300 cycles with a high capacity retention of 76.02%. The high‐voltage ASSLMB also shows negligible capacity degradation after 3000 bending cycles with a bending radius of 4.0 mm. This work provides a new insight into the effective design strategy of oxidation‐stable polyether electrolytes for the long‐term operation of high‐voltage LMBs.

## 2 Results and Discussion

### 2.1 Design Principle and Fabrication of Difluorinated Polyether Electrolytes

**Figure 1:**
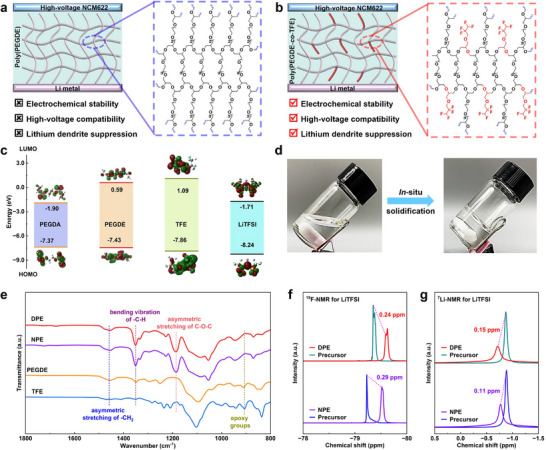
Design principle and physicochemical characterizations of difluorinated polyether electrolytes. a,b) Schematic illustration of NPE and DPE in high‐voltage LMBs. c) The HOMO and LUMO energy levels of PEGDA, PEGDE, TFE and LiTFSI. d) Digital photographs showing in situ polymerization of polymer electrolytes. e) FTIR spectra of PEGDE, TFE, NPE and DPE. f) ^19^F‐NMR spectra of LiTFSI in NPE, DPE and their precursors. g) ^7^Li‐NMR spectra of NPE, DPE and their precursors.

Two SPEs, named DPE and NPE, were prepared by one‐step in‐situ thermal polymerization using PEGDE with or without TFE as the monomer, LiTFSI as Li salt, and LiBF_4_ as an initiator (**Figure** **1**a,b). Compared with NPE, DPE has high oxidation stability, dendrite inhibition ability, and excellent ionic conductivity. The synthesis mechanism of DPE can be attributed to the cationic ring‐opening copolymerization. The added LiBF_4_ can be decomposed into LiF and BF_3_ under heating conditions. Subsequently, BF_3_ can bind with trace amounts of H_2_O to generate H^+^(BF_3_OH) complexes, and simultaneously attack the oxygen atoms of the epoxy groups of both monomers, thereby initiating cationic ring‐opening copolymerization.^[^ ^39^, ^40^ ^]^ In principle, an inherently stable SPE should have lower highest occupied molecular orbitals (HOMO) and higher lowest unoccupied molecular orbitals (LUMO), indicating its better stability for high‐voltage cathodes and Li metal anodes.^[^ ^36^, ^41^ ^]^ Density functional theory (DFT) calculations reveal that the LUMO energy level of PEGDE is much higher than that of commonly used polyethylene glycol diacrylate (PEGDA), indicating its excellent stability for Li metal anodes (Figure 1c). In addition, the HOMO energy level of PEGDE is also slightly lower than that of PEGDA, indicating that it has better oxidation stability. Therefore, cyclic fluorinated ether has been selected to improve the interfacial stability of PEGDE by introducing TFE chain segments due to the strong electron‐withdrawing effect of fluorine functional groups.

To confirm the successful polymerization of NPE and DPE, Fourier transform infrared spectroscopy (FTIR) and nuclear magnetic resonance (NMR) were conducted to analyze the chemical structures. The original liquid precursor of PEGDE and TFE is transformed into a transparent solid electrolyte after heating from the digital images in Figure 1d. As shown in Figure 1e, the FTIR spectra show the epoxy characteristic peaks of PEGDE and TFE precursors at 911 cm^−1^. After polymerization, the corresponding epoxy group peaks in NPE and DPE disappear. The new peak appearing at 1190 cm^−1^ is attributed to the asymmetric stretching of C─O─C bonds in the polymerized segment. Compared with the precursors of PEGDE and TFE, NPE and DPE exhibit sharper C─H bending vibration peaks at 1350 cm^−1^ and asymmetric stretching peaks of −CH_2_ at 1470 cm^−1^. The peak near 1100 cm^−1^ belongs to the symmetric stretching of C−O−C in PEGDE and TFE. The peaks of NPE and DPE shift to 1050 cm^−1^, indicating a strong interaction between Li^+^ and EO groups. The above results demonstrate the successful open‐ring polymerization of PEGDE and TFE. In addition, the measurements of ^13^C‐NMR (Figure S1, Supporting Information) and ^1^H‐NMR (Figure S2, Supporting Information) also confirm the successful polymerization. Based on ^1^H‐NMR spectra, the polymerization conversion rate of DPE has been estimated to be 96.5%. The high polymerization conversion rate of DPE is beneficial for reducing interface side reactions between residual precursors and electrodes. Furthermore, the thermal properties of NPE and DPE were investigated through TGA and DSC measurements (Figure S3, Supporting Information). The decomposition temperature of DPE declined to 175.9 °C after grafting TFE into NPE. This phenomonen is attributed to the decreased cross‐linking degree, which weakens the molecular interaction between polymer chains and facilitates the molecular motion at high temperatures. The glass trasition temperatures (T_g_) was measured to be ‐53.71 and ‐54.73 °C for NPE and DPE, respectively. The lower T_g_ of DPE implies faster Li^+^ transportation and higher ionic conductivity.

The coordination between Li^+^ cations, TFSI^−^ anions and fluorinated polymers was characterized by ^19^F‐NMR and ^7^Li‐NMR spectra. As shown in Figure 1f, both NPE and DPE exhibits notable upfield shifts in ^19^F‐NMR spctra, indicating enhanced interactions between TFSI^−^ anions and the two polymers after polymerization.^[^ ^42^ ^]^ Compared with NPE, DPE shows a much larger upfield shift, suggesting stronger coordination with TFSI^−^ anions. The enhanced interactions between TFSI^−^ anions and fluorinated polymers are beneficial for suppressing anion mobility. Raman spectra was conducted to further confirm the changes of solvation structure after grafting TFE into NPE (Figure S4, Supporting Information). The peaks around 737, 741, and 747 cm^−1^ correspond to free TFSI^−^ anions, contact ion pairs (CIP) and aggregates (AGG), respectively.^[^ ^43^, ^44^ ^]^ The AGG propotion in DPE increased to 15.04% from 6.10% in NPE. The free TFSI^−^ anions show marked decline in DPE, indicating enhanced interaction between TFSI^−^ anions and Li^+^. The interaction between Li^+^ cations and fluorinated polymers was also well studied by ^7^Li‐NMR (Figure 1g). Compared to the polymer precursors, DPE shows larger downshifts (0.15 ppm) than NPE (0.11 ppm), indicating the increased interaction of Li^+^ cations and fluorinated polymer skeletons. This enhancement is attributed to the incorporation of difluorinated TFE segments. In liquid fluorinated electrolytes, difluorinated substituents are coordinated with Li^+^ cations.^[^ ^45^ ^]^ The Li−F interaction was also supported by the difference in chemical shifts of TFE with and without Li salts (Figure S5, Supporting Information). Therefore, the difluorination design may promote the migration of Li^+^ ions by coordinating with Li^+^ cations and TFSI^−^ anions in fluorinated polymer electrolytes.

### 2.2 Electrochemical Properties of Difluorinated Polyether Electrolytes

**Figure 2:**
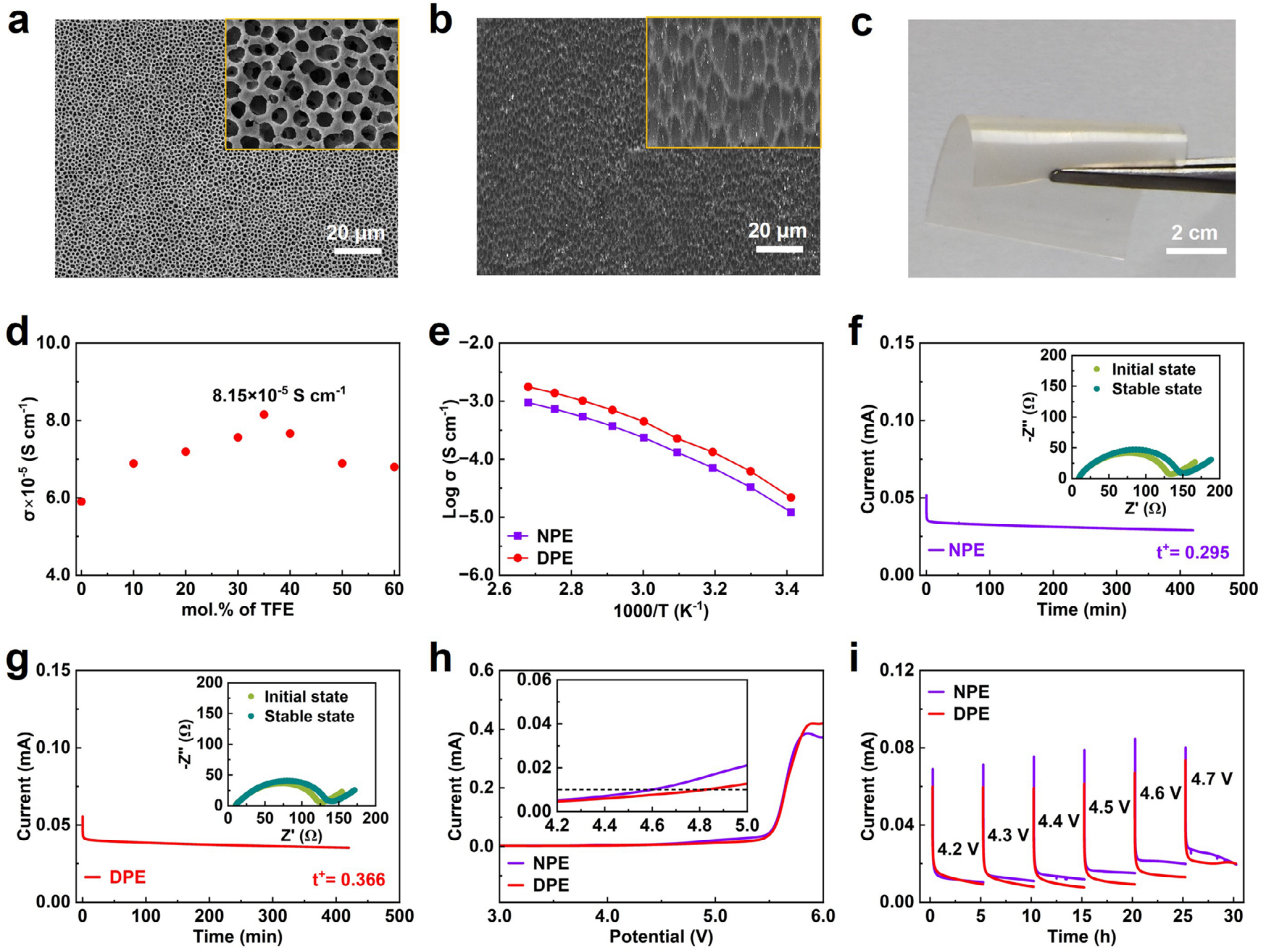
Electrochemical properties of difluorinated polyether electrolytes. a) SEM images of (a) fluorinated mesoporous film and (b) DPE within the fluorinated mesoporous film. c) Digital photograph of DPE after in‐situ polymerization. d) Optimization of ionic conductivity with different ratios of TFE. e) Ionic conductivities at the temperature range of 20–100 °C. Polarization curves of f) NPE and g) DPE. Inset graphs are the Nyquist plots before and after the polarization test. h) LSV profiles for both polymer electrolytes at 0.5 mV s^−1^. i) Electrochemical floating analysis of Li||NCM622 cells with NPE and DPE.

To reduce the interfacial resistance of solid‐state electrolytes, a mechanically robust fluorinated polyether electrolyte film was prepared by infiltrating PEGDE and TFE precursor solutions into a vertical mesoporous polymer matrix and initiating in‐situ thermal polymerization. Here, a mechanically robust mesoporous poly(vinylidene fluoride‐co‐hexa‐fluoropropylene) (PVDF‐HFP) film was prepared as the electrolyte host by the slurry coating and solvent evaporating process. Scanning electron microscope (SEM) characterization indicates the pore size of mesoporous film is controlled at 2 µm and evenly distributed (**Figure** **2**a). After in‐situ thermal polymerization, DPE can be filled into all vertical pore structures of the mesoporous film (Figure 2b; Figure S6, Supporting Information). The fluorinated polyether electrolyte shows a film thickness of 12 µm similar to the original mesoporous film and commercial separator (Figure S7, Supporting Information). Importantly, the solid‐state Li||Li symmetric cell using the mesoporous film delivers the lowest bulk and interfacial resistance compared to commercial separators (Figures S8 and S9, Supporting Information). In addition, the resistance of stainless steel (SS)||SS symmetric cells is almost unchanged after 8 h thermal polymerization (Figure S10, Supporting Information). The as‐prepared freestanding film of fluorinated polyether electrolyte exhibits a transparent appearance and excellent flexibility (Figure 2c). Benefiting from the excellent mechanical robustness and interfacial contacts, the fluorinated polyether electrolytes are promising for ASSLMBs.

The ion conductivity and transference number of fluorinated polyether electrolytes are evaluated as two critical performance metrics for ASSLMBs. The ionic conductivities of DPE were measured by changing the addition ratios of TFE at room temperature (Figure 2d). The ion conductivity gradually increases with the addition of TFE and then reaches its maximum value of 8.15×10^−5^ S cm^−1^ at an optimal addition amount of 35%. The proper addition of TFE could promote Li^+^ transport by enhancing the interaction of TFSI^−^ anions with polymer chains and reducing the polymer crosslinking crystalline regions. However, the excessive addition of TFE also sacrifices the ion conductivity of DPE due to the reduced ion‐conducting EO groups.^[^ ^35^, ^46^ ^]^ Compared to NPE, the Young's modulus of DPE declined to 19.67 MPa from 30.17 Mpa after introducing TFE side chains (Figure S11, Supporting Information). The ion conductivities of DPE and NPE are also recorded within the wide temperature ranges from 20 to 100 °C, which is fitted by the Vogel‐Fulcher‐Tamman equation. Due to the TFE introduction, DPE shows much higher ionic conductivity than NPE over the whole temperature range of 20–100 °C (Figure 2e). In particular, a high ion conductivity of 2.0×10^−4^ S cm^−1^ can be obtained at 50 °C for DPE. The Vogel‐Fulcher‐Tamman fitting results demonstrate no significant change in activation energy (0.28 eV) for these two electrolytes. The Li^+^ transference number of NPE and DPE was measured through chronoamperometry combined with electrochemical impedance spectroscopy (Figure 2f,g). The DPE shows a much higher Li^+^ transference number (0.366) than that (0.295) of NPE due to the hydrogen interactions between TFSI^−^ anions and fluorinated polymers.^[^ ^47^, ^48^ ^]^

The electrochemical stability window of polymer electrolytes is another important indicator for determining their potential for matching with high‐voltage cathodes. Linear sweep voltammetry (LSV) measurements of Li||SS cells were used to monitor the electrochemical stability window of NPE and DPE at a standard leakage current of 10 µA. Benefiting from the strong electron‐drawing effect of TFE segments, DPE exhibits a much higher oxidation potential (4.83 V) than that (4.59 V) of NPE. To examine the true oxidation stability of fluorinated polyether electrolytes on high‐voltage cathode, potentiostatic polarization measurements were also conducted on Li||NCM622 cells using two types of polymer electrolytes to detect the oxidation leakage current (Figure 2i). The Li||NCM622 cell with DPE shows the smallest leakage current under various applied voltages ranging from 4.2 to 4.7 V. In contrast, the NPE‐based cell shows a much larger leakage current after charging at 4.3 V, indicating serious oxidation decomposition reactions under high‐voltage conditions.

### 2.3 Interfacial Stability of Difluorinated Polyether Electrolytes

**Figure 3:**
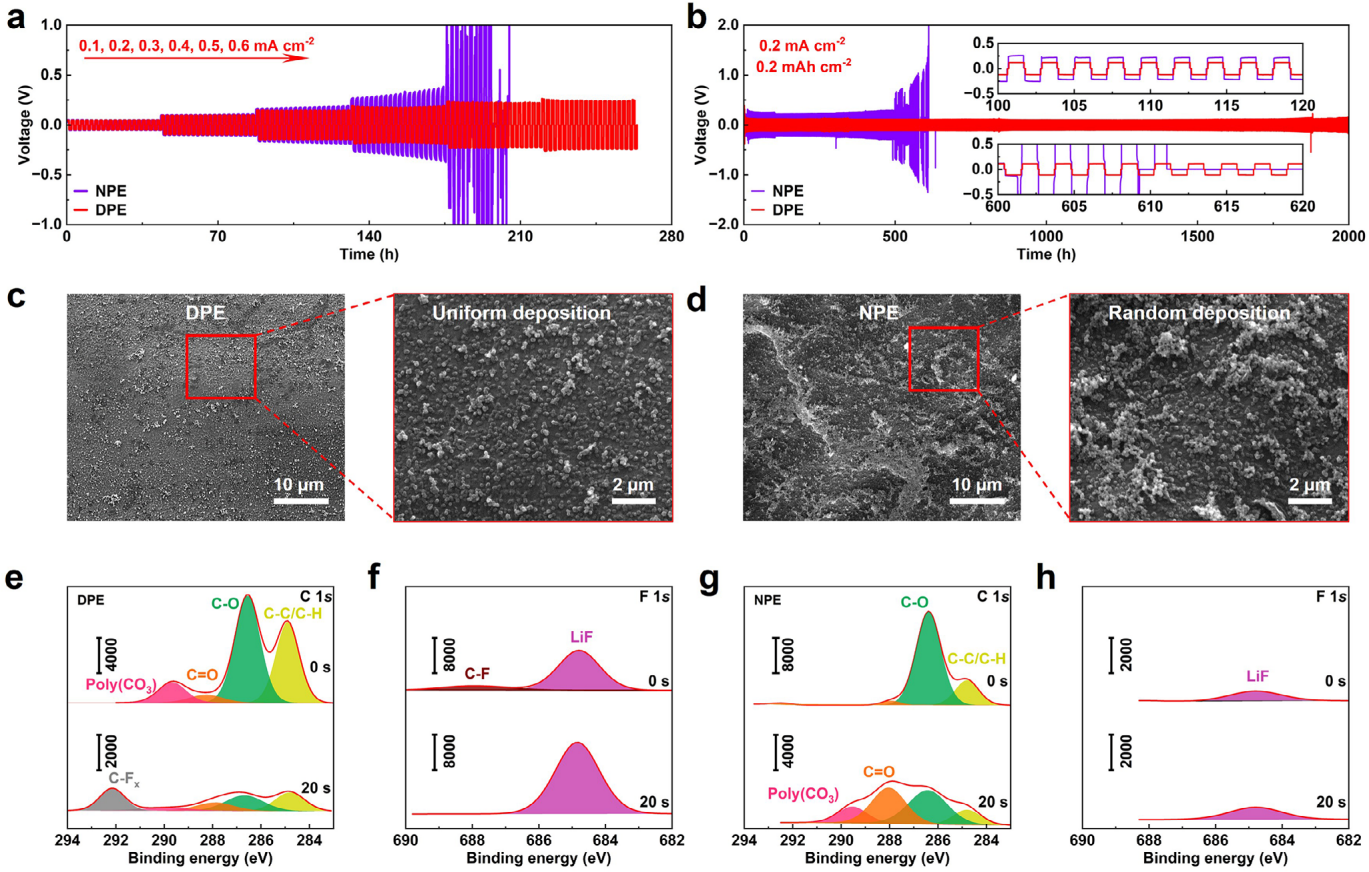
Interfacial stability of difluorinated polyether electrolytes against Li metal anode. a) Rate performance of Li||Li batteries at different current densities from 0.1 to 0.6 mA cm^−2^ with step‐time of 1.0 h at 50 °C. b) Cycling performance of Li||Li batteries at 0.2 mA cm^−2^. SEM images of deposited Li metal with c) DPE and d) NPE after 20 cycles. XPS spectra with etching depth profiles of (e, f) C 1*s* and (g, h) F 1*s* after 20 cycles.

The electrochemical stability against Li metal anode affects the lifetime of battery cycling performance. Li metal plating/stripping behavior was investigated in Li||Li symmetric cells with NPE and DPE at various current densities (**Figure** **3**a). Benefiting from the formation of a stable solid electrolyte interface (SEI), the Li|DPE|Li cell maintains a stable Li plating/stripping behavior at a high current density of 0.6 mA cm^−2^. In sharp contrast, a large voltage oscillation occurs at 0.3 mA cm^−2^ for Li|NPE|Li cell. Further, an increase in current density to 0.5 mA cm^−2^ results in a rapid decrease in voltage and a short circuit, indicating the formation of an unstable SEI within NPE.^[^ ^49^ ^]^ Long‐term cycling performance was evaluated at 0.2 mA cm^−2^, as shown in Figure 3b. The Li|DPE|Li cell exhibits stable Li plating/stripping for 2000 h without voltage fluctuation, indicating effective Li dendrite suppression. While the Li|NPE|Li cell experiences a significant increase in voltage at 500 h, followed by an internal short circuit after 600 h. The alternating current impedance of symmetrical cells also confirms the superior stability of DPE (Figure S12, Supporting Information). The resistance for Li|NPE|Li cell continuously increases over cycling, attribured to side reactions between NPE and Li metal anode. In comparison, the Li|DPE|Li cell demonstrates relatively stable resistance after 5 cycles. Moreover, the Li|DPE|Li sustains stable plating/stripping behavior for up to 750 h at a higher current density of 0.3 mA cm^−2^ (Figure S13, Supporting Information). The above results confirm that DPE enables robust Li plating/stripping behavior during long‐term cycling processes.

SEM imaging was conducted to further compare the morphology differences of Li metal in NPE and DPE during the plating/stripping process (Figure 3c). The cycled Li metal with DPE shows uniform nanosphere‐like deposition after 20 cycles at 0.2 mA cm^−2^. In comparison, a uneven and random deposition was observed in NPE (Figure 3d). In addition, a more dense structure of deposited Li metal particles was obtained with DPE for a capacity loading of 1.0 mAh cm^−2^, while loosely packed deposits accompanied by large voids are seen in NPE (Figure S14, Supporting Information). Apart from the effect of DPE, the fluorinated mesoporous electrolyte film can also redistribute the Li^+^ flux through the strong interactions between polar fluorinated groups and Li metal. The aligned F atom in fluorinated mesoporous film enhances the interaction of C−F and Li metal, promoting uniform Li metal deposition.^[^ ^50^ ^]^ X‐ray photoelectron spectroscopy (XPS) characterization was further performed to reveal the interfacial composition of the cycled Li metal anode of symmetric cells with DPE and NPE (Figure 3e–h). In C 1*s* depth profiles, the interface of both polymer electrolytes shows four characteristic peaks of C−C/C−H (284.8 eV), C−O (286.5 eV), C═O (288.1 eV) and poly (CO_3_) (289.9 eV), which is accorded with reported polyether electrolytes.^[^ ^51^ ^]^ The C 1*s* depth profiles indicate the interface of DPE has much fewer organic components than NPE at various etching depths. In addition, the F 1*s* depth profiles of both polymer electrolytes reveal that the LiF inorganic components are dominant in the interface of DPE, which is well contrasted with NPE (Figure S15, Supporting Information). The LiF component keep electrochemical stability with Li metal anode and high‐voltage cathode due to its lithiophobic nature, weak bonding, and high interfacial energy with Li metal.^[^ ^52^ ^]^ Therefore, the LiF‐rich interface of DPE promotes stable Li plating/stripping behaviors.

**Figure 4:**
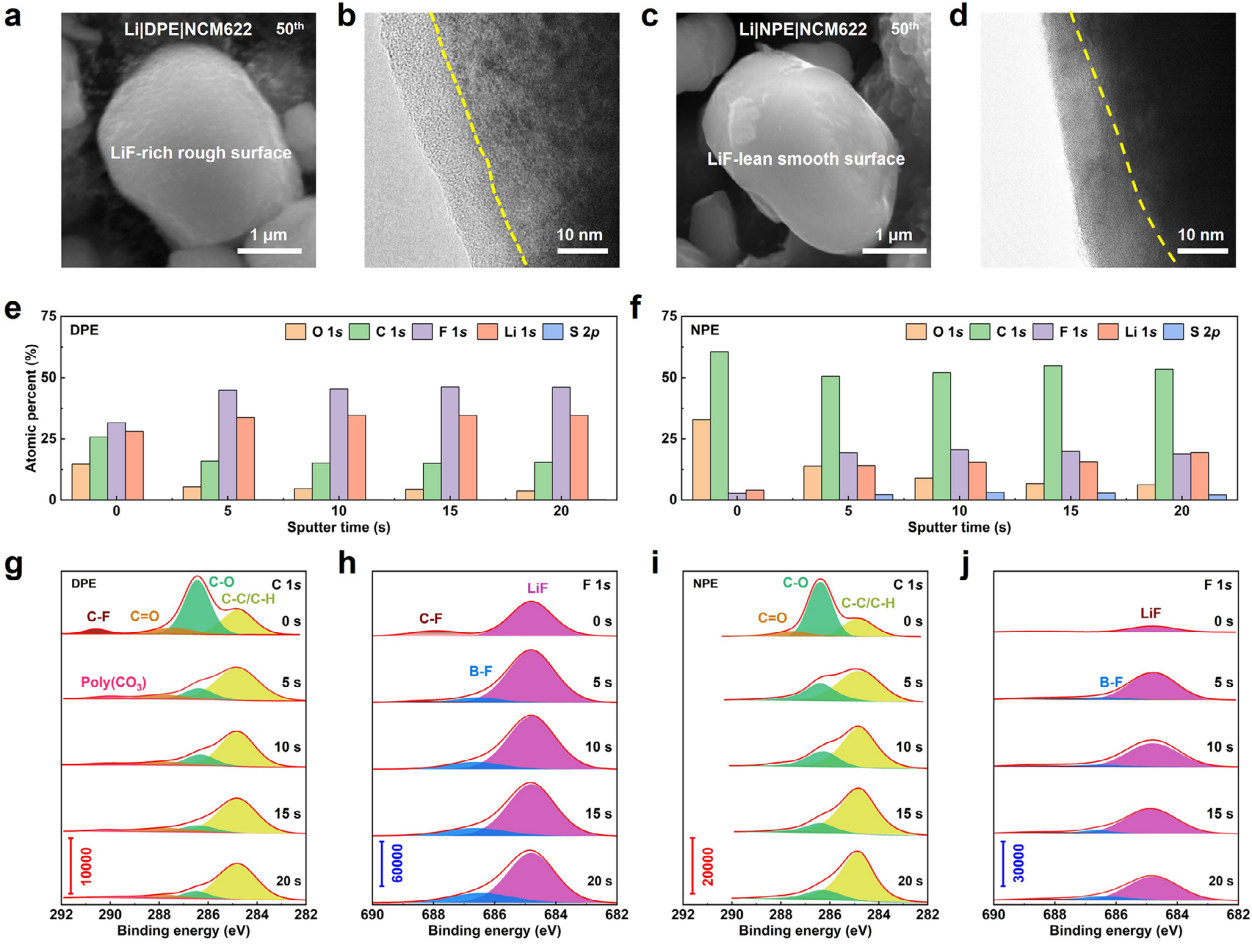
Interfacial stability of difluorinated polyether electrolytes against high‐voltage cathode. a,c) SEM and b,d) TEM images of cycled NCM622 with DPE and NPE. XPS elemental depth profiles of cycled NCM622 with e) DPE and f) NPE. g,i) C 1*s* and h,j) F 1*s* XPS spectra of cycled NCM622 with DPE and NPE after etching 5, 10, 15, and 20 s.

In addition to the interface of the Li metal anode, a stable cathode electrolyte interphase (CEI) of DPE is also required to match the high‐voltage cathode. The CEI components on NCM622 after 50 cycles were examined by SEM and high‐resolution transmission electron microscopy (TEM). As shown in **Figure** **4**a, SEM image shows a rough yet uniform LiF‐rich interlayer on the surface of particle in cycled NCM622 with DPE, which is beneficial for mitigating cracking during cycling. TEM image further confirms the formation of a uniform CEI layer ≈8 nm in thickness (Figure 4b). In contrast, a smoother coating interface is observed for the cycled NCM 622 particle in NPE, while TEM image reveals an uneven and significantly thicker CEI layer, indicative of excessive decomposition (Figure 4c,d). Elemental analysis based on SEM results preforms that the fluorinated groups in DPE promote the formation of a LiF‐rich CEI layer on the NCM622 cathode, contributing to improved interfacial stability (Table S1, Supporting Information).

The CEI component of DPE was also studied by XPS characterization after 50 cycles of high‐voltage cathode. Etching depth profiles are used to evaluate the component distribution in the CEI layer on cycled NCM622 particles along with the depth (Figure 4e,f). The CEI layer of DPE shows a steady atomic percentage of C and F, while the atomic percentage of C and F in NPE varies randomly through etching depth. The inorganic components in DPE is much higher than in NPE. This result indicates a uniform inorganic‐rich CEI layer is formed on the surface of the NCM622 cathode with DPE. In C 1*s* depth profiles, both DPE and NPE samples show three common peaks of C−C/C−H (284.8 eV), C−O (286.5 eV), and C═O (288.1 eV) (Figure 4g,i). It is worth noting that two additional signal peaks of poly (CO_3_) (290.1 eV) and C−F (290.6 eV) are detected at the CEI interface of DPE, attributing to the decomposition of TFE segments. In F 1*s* spectra, a certain amount of inorganic Li*_x_* BO*_y_* F*_z_* (BF) and LiF is observed at the peak position of 686.5 and 684.8 eV for both polymer electrolytes (Figure 4h,j). However, the intensity of inorganic components in DPE is double higher than in NPE, which is ascribed to the anion‐rich solvation structure of DPE resulting from the electron‐withdrawing effect of TFE. High AGG proportion in DPE contributes to anion‐derived and LiF‐rich CEI. The intensity of organic or inorganic species remains stable along the etching depth, consistent with the depth profiles of CEI in Figure 4f. The above results demonstrate that the CEI layer of DPE is inorganic‐rich components, while organic components are dominant for NPE.

### 2.4 Electrochemical Performance of ASSLMBs with Difluorinated Polyether Electrolytes

**Figure 5:**
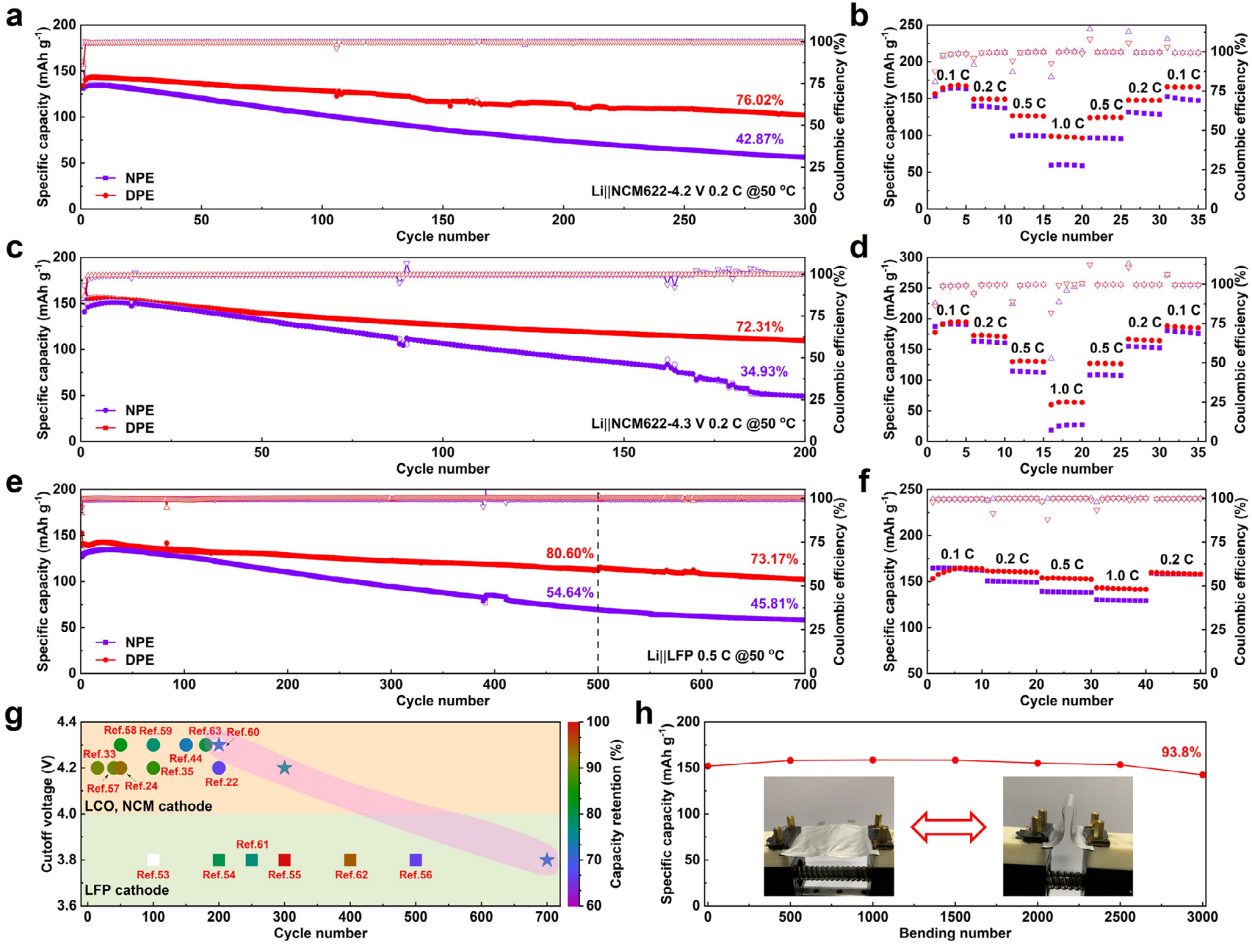
Electrochemical performance of ASSLMBs with difluorinated polyether electrolytes. a,b) Cycling stability and rate performance of Li||NCM622 cells with the cut‐off voltage of 4.2 V. c,d) Cycling stability and rate performance Li||NCM622 cells with the cut‐off voltage of 4.3 V. e,f) Cycling stability and rate performance Li||LFP cells. g) Comparison of comprehensive battery performance with reported studies using polyether‐based electrolytes. h) Flexibility of assembled solid‐state pouch cell under the radius of 4.0 mm.

To evaluate the oxidation stability of various polyether electrolytes, the electrochemical performances of ASSLMBs were assessed by pairing DPE with Li metal anode and high‐voltage LiNi_0.6_Co_0.2_Mn_0.2_O_2_ (NCM622) cathode at a cut‐off voltage of 4.2 V. As results, the Li|DPE|NCM622 cell delivers an initial discharge capacity of 134.19 mAh g^−1^ with an initial Coulombic efficiency (ICE) of 87.57% at 0.2 C, significantly higher than that of Li|NPE|NCM622 cells (80.89%) (**Figure** **5**a). Following a five‐cycle activation process, the Li|DPE|NCM622 cell maintains a high discharge capacity of 142.95 mAh g^−1^ over 300 cycles, corresponding to a capacity retention of 76.02%, markedly outperforming the Li|NPE|NCM622 cells. In terms of rate capability, the Li|DPE|NCM622 cell consistently exhibits higher specific capacities across a broad range of current densities from 0.1 C to 1.0 C. Specially, the initial discharge capacity at 0.1 C is 156.64 mAh g^−1^, which increases to 167.31 mAh g^−1^ after three cycles. As the current density changes, discharge capacities of 149.5, 126.5, and 98.92 mAh g^−1^ were obtained at 0.2 C, 0.5 C, and 1.0 C, respectively. Upon returning to 0.1 C, the discharge capacity recovers to 165.83 mAh g^−1^, confirming excellent rate capability (Figure 5b). In comparison, the Li|NPE|NCM622 cell performs a maximum discharge capacity of 153.69 mAh g^−1^ at 0.1 C, but only recovers to 147.43 mAh g^−1^ after returning to the same rate. These results clearly demonstrate the enhanced cycling stability and rate capability of DPE compared to NPE.

The oxidation stability of DPE was further assessed by increasing the cut‐off voltage of the NCM622 cathode to 4.3 V (Figure 5c). The Li|DPE|NCM622 cell shows a high initial discharge capacity of 151.23 mAh g^−1^ with an ICE of 86.86%. While the Li|NPE|NCM622 cell barely exhibits a discharge capacity of 140.78 mAh g^−1^ and an ICE of 83.15%. Notably, the CE of Li|DPE|NCM622 cell increases up to 99.10% after the second cycle, whereas the Li|NPE|NCM622 cell reaches only 97.16% (Figure S16, Supporting Information). This indicates that DPE enables solid‐state batteries to reach a stabilization earlier than NPE. At 0.2 C, the Li|DPE|NCM622 cell retains 72.31% of its capacity after 200 cycles, significantly higher than the 34.93% retention observed for Li|NPE|NCM622 cell. Similar improvements are also reflected in rate capability at a rates ranging from 0.1 C to 1.0 C, where discharge capacities of 194.79, 173.08, 131.15, and 64.34 mAh g^−1^ at 0.1, 0.2, 0.5, and 1.0 C, respectively, were obtained in Li|DPE|NCM622 cell (Figure 5d). In particular, a recovered discharge capacity of 188.09 mAh g^−1^ after returning to 0.1 C was collected in Li|DPE|NCM622 cell, indicating the remarkable rate performance. In contrast, the discharge capacity of Li|NPE| NCM622 cell can only recover to 180.67 mAh g^−1^ at 0.1 C after the rate measurement. It is worth noting that the dispartity in capacity between these two solid‐state electrolytes becomes more pronounced at high current density, especially at 1.0 C, which correlates with reduced polarization and improved interfacial stability of DPE with enhanced ions mobilty and ionic conductivity under fast Li^+^ transport conditions.

The Li|DPE|LFP cell was also assembled to evaluate the rate capability of DPE in ASSLMB (Figure 5e). At a high rate of 0.5 C, the capacity retention of Li|DPE|LFP cell is 80.60% and 73.17% after 500 cycles and 700 cycles, respectively. In contrast, the capacity retention of Li|NPE|LFP cell is only 45.81% after 700 cycles. In addition, the DPE‐based cell also exhibits much higher specific capacities than NPE‐based cells at various rates (Figure 5f). These results confirm that DPE enables both excellent rate capability and long‐term cycling stability, demonstrating its outstanding compatibility in solid‐state battery system used Li metal anode and high‐voltage cathodes under particle operating conditions.

To highlight the comparable oxidation stability of fluorinated polyether electrolytes with the high‐voltage cathode, the cut‐off voltage, cycle number and capacity retention of our ASSLMBs were selected to compare with previously reported LMBs with polyether electrolytes (Figure 5g). The Li|DPE|LFP battery exhibits the best cycling stability among these devices.^[^ ^53^, ^54^, ^55^, ^56^ ^]^ Furthermore, the high‐voltage Li|DPE|NCM622 battery operates stably for 300 cycles at 4.2 V and 200 cycles at 4.3 V, respectively, with capacity retention values exceeding those of previously reported counterparts under similar conditions.^[^ ^22^, ^24^, ^33^, ^35^, ^44^, ^57^, ^58^, ^59^, ^60^, ^61^, ^62^, ^63^ ^]^ Impressively, a large‐size flexible Li|DPE|NCM622 pouch cell was constructed with textile composite electrodes (Li anode and NCM622 cathode), which are fabricated as our previous works.^[^ ^64^, ^65^, ^66^ ^]^ As shown in Figure 5h, this flexible pouch cell shows a high capacity retention of 93.8% after 3000 bending cycles with a radius of 4.0 mm. The charge‐discharge profiles reveal a slight capacity decline from the initial 151.96 to 142.52 mAh g^−1^ after 3000 bending cycles, confirming both electrochemical and mechanical robustness (Figure S17, Supporting Information). Therefore, the fluorinated polyether electrolyte not only enhances compatibility with high‐voltage cathode to provide higher energy density but also broadens the application in all‐solid‐state flexible Li batteries.

## 3 Conclusion

We have proposed an effective strategy to enhance the compatibility with high‐voltage LMBs using difluorinated polyether electrolytes. Due to the synergistic electron‐withdrawing and ion solvation effects of difluorinated functional groups, the designed DPE shows an improved oxidation potential of 4.9 V (vs Li^+^/Li) and ionic conductivity (2.0 × 10^−4^ S cm^−1^), which is superior to non‐fluorinated polyether electrolyte (NPE). Moreover, the generated LiF‐rich electrolyte/electrode interphase ensures the stability of DPEs against both Li metal anode and high‐voltage cathode. The Li||Li symmetric cell with DPE can be stably operated for over 2000 h with an extremely small overpotential of 20 mV. The assembled all‐solid‐state Li||LFP battery with DPE shows long‐term cycling stability with a high capacity retention of 73.17% after 700 cycles. The high‐voltage all‐solid‐state Li||NCM622 battery can be stably operated for 300 cycles with a high capacity retention of 76.02%. The high‐voltage ASSLMB also shows negligible capacity degradation after 3000 bending cycles with a bending radius of 4.0 mm. These results collectively demonstrate that molecular‐level fluorination of polyether backbones significantly improves both the intrinsic stability of the electrolyte and its interfacial compatibility, offering a promising route for the development of high‐performance ASSLMBs.

## 4 Experimental Section

### Preparation of Difluorinated Polyether Electrolytes

0.25 g LiTFSI (99.5%, Capchem) and 0.02 g LiBF_4_ (Initiator: 2.0 wt.%, 99.9%, Aladdin) were dissolved into 1.0 g PEGDE (Mn = 500, Aladdin) or mixed solution of 1.0 g PEGDE with corresponding molar ratio of TFE. After stirring to obtain the homogeneous solution, the precursors were poured into the PTFE holders with sealed lid. Sebquently, they were heated at 60 °C for 8 h to get the self‐supporting NPE and DPE films.

### Preparation of Fluorinated Mesoporous Film

Lithium nitrate (LiNO_3_) and polyvinylidene fluoride‐hexafluoropropylene (PVDF‐HFP) were dissolved into acetone (ACN) and stirred until a homogenous solution. The weight ratio of ACN, LiNO_3,_ and PVDF‐HFP is 90:5:5. Subsequently, the solution was blade‐casted onto the aluminum‐plastic film. After solvent evaporation, the semi‐transparent fluorinated mesoporous film was peeled from the aluminum–plastic film and dried in the vacuum oven overnight at 80 °C. The porosity of the porous PVDF‐HFP film is around 70.5%.

### Assembly of Solid‐State Lithium Batteries

The solid‐state batteries were assembled with CR2025‐type coin cells by contacting lithium metal, SPEs, and cathodes in an argon‐filled glove box. For in‐situ assembly, the fluorinated mesoporous film was used to host precursors of fluorinated polyether electrolytes. The assembled batteries are placed in an oven for 8 h at 80 °C before testing. The NCM622 cathode was composed of 70 wt% NCM_622_, 10 wt% Super‐P, 10 wt% PVDF, and 10 wt% plasticizer (0.5 M LiFSI in IL), dissolving into N‐methyl pyrrolidone (NMP). Then the slurries were coated onto carbon‐coated aluminum film. The LFP cathode was prepared by dissolving LFP, Super‐P, binder (PEO and LiClO_4_, EO: Li^+^ = 18:1) (70:10:20) into anhydrous acetonitrile. Subsequently, the LFP cathode and NCM622 cathode were dried overnight in a vacuum oven at 60 and 100 °C, respectively. The mass loading of the cathodes is around 2.0 mg cm^−2^.

### Material Characterization

Proton nuclear magnetic resonance (^1^H‐NMR), carbon nuclear magnetic resonance (^13^C‐NMR), and fluorine nuclear magnetic resonance (^19^F‐NMR) were conducted on a Bruker 400 MHz spectrometer. For NMR samples, CDCl_3_ was chosen to lock fields and the measurements adopt decoupling mode. Fourier transform infrared spectra (FTIR, Nicolet iS50) were performed to characterize the structure of raw materials and SPEs over the range of 4000–500 cm^−1^. Thermogravimetric analysis (TGA, Mettler‐Toledo) was conducted with a heating rate of 10 °C min^−1^ from 25–600 °C under a nitrogen atmosphere. The glass transition temperature (T_g_) was characterized by differential scanning calorimetry (DSC, Mettler‐Toledo) with sealed Al pan hosting samples of 5–10 mg. All the samples were cooled to ‐60 °C first and then heated to 100 °C with a cooling and heating rate of 5 °C min^−1^. The surface morphology of all the samples was characterized with field‐emission scanning electron microscopy (FE‐SEM, Tescan MIRA3). The components of the sample surface were analyzed using X‐ray photoelectron spectroscopy (XPS, PHI5000 Versaprobe III) with a monochromatic Al‐Kα (1486.6 eV) radiation source. The XPS spectra are calibrated by using C 1*s* (284.8 eV) as the reference peak. The samples of cycled lithium metal and cathode were immersed into dimethyl carbonate (DMC) solvent for 24 h and washed three times to remove residual SPEs. The microscopy of the cycled NCM622 cathode was characterized by transmission electron microscopy (TEM, Talos F200X G2).

### Electrochemical Measurements

All the electrochemical performances were conducted with CR2025‐type coin cells, assembled with fluorinated mesoporous film hosting precursors. The assembled batteries are placed in an oven for 8 h at 80 °C. And then the electrochemical performance was tested at 50 °C. The ionic conductivities, liner sweep voltammograms (LSV), lithium‐ion transference number (*t^+^*), and electrochemical floating analysis were measured with a Solartron multi‐channel potentiostat electrochemical workstation (1260 A). The ionic conductivities were measured by electrochemical impedance spectroscopy (EIS) using the perturbation signal of 10 mV over the frequency of 1 MHz to 10 Hz. The SPEs were sandwiched with stainless steel blocking electrodes with SS|SPEs|SS configuration. The thickness of SPEs was equal to that of fluorinated mesoporous film. The ionic conductivity was calculated by the following Equation (1)

(1) $$\sigma =\frac{L}{S\cdot R}$$

where *L*(cm) stands for the thickness of SPEs, *S* (cm^2^) stands for the contact area of SS and SPEs, and *R* (Ω) represents the bulk resistance of blocking batteries obtained by EIS. The electrochemical stable window of SPEs was evaluated by LSV over the potential of 0–6.0 V with a scanning rate of 0.5 mV s^−1^ at 50 °C, using Li|SPEs|SS configuration. The lithium‐ion transference number was investigated with the combination of AC impedance and DC polarization, using a symmetrical blocking electrode of Li|SPEs|Li configuration. AC impedance of symmetrical cells was detected over the frequency of 1 MHz to 0.01 Hz by EIS. The voltage of DC polarization is 10 mV. The values of *t^+^* were calculated by Bruce–Vincent‐Evans Equation (2)

(2) $$t^{+}=\frac{I_{ss}\left(\Delta V-I_{0}R_{0}\right)}{I_{0}\left(\Delta V-I_{ss}R_{ss}\right)}$$

where *I_0_* (A) and *I_ss_* (A) represent the initial current and the stable current after polarization, respectively. ∆V (V) is the applied voltage of DC polarization. *R_0_* (Ω) and *R_ss_* (Ω) stand for the interfacial resistance before and after polarization, respectively. The electrochemical stability of SPEs against lithium was tested on the NEWARE battery testing system through galvanostatic cycling measurements at the constant current density, using symmetrical cells of Li|SPEs|Li configuration. The cells were charged and discharged for 1.0 h. The battery performance of cells paring with LFP cathode was tested within the potential of 3.0–3.8 V (vs Li^+^/Li) (1.0 C = 170 mAh g^−1^). The Li|SPEs|NCM622 cells were charged to 4.2 or 4.3 V (vs Li^+^/Li) and discharged to 3.0 V (vs Li^+^/Li) (1.0 C = 180 mAh g^−1^).

## Conflict of Interest

The authors declare no conflict of interest.

## Supporting information



Supporting Information

## Data Availability

The data that support the findings of this study are available in the supplementary material of this article.
