# Supplementary material for: Molecular Design of Difluorinated Polyether Electrolyte for Ultrastable High‐Voltage All‐Solid‐State Lithium Metal Batteries
Source: Adv Sci (Weinh). 2025 Aug 11;12(41):e08721. doi: 10.1002/advs.202508721 (PMC12591110; doi:10.1002/advs.202508721)
Supplement: Supplementary file 1 — Supporting Information [file ADVS-12-e08721-s001.docx]

Supporting Information

**Molecular Design of Difluorinated Polyether Electrolytes for High-Voltage All-Solid-State Lithium Metal Batteries**

*Zhenyao Wei, Yufeng Luo, Yongqiang Yang, Yaxin Tang, Junhua Zhou, Chao Luo, Ruo Wang, Huipeng Zeng, Chaoyang Wang, Xiaoxiong Xu, Yonghong Deng*, Zijian Zheng* and Jian Chang**
Dr. Z. Wei, Dr. Y. Tang, Dr. C. Luo, Dr. R. Wang, Mr. Zeng, Prof. X. Xu, Prof. Y. Deng
Department of Materials Science and Engineering
Guangdong Provincial Key Laboratory of Energy Materials for Electric Power
Southern University of Science and Technology
Shenzhen, 518055, China.
Email: [dengyh@sustech.edu.cn](mailto:dengyh@sustech.edu.cn)

Dr. Z. Wei, Prof. Z. Zheng
School of Fashion and Textiles
The Hong Kong Polytechnic University
Hong Kong SAR, China
Email: [tczzheng@polyu.edu.hk](mailto:tczzheng@polyu.edu.hk)

Dr. Y. Luo, Dr. Y. Yang, Dr. J. Zhou, Prof. Z. Zheng
Department of Applied Biology and Chemical Technology
The Hong Kong Polytechnic University
Hong Kong SAR, China

Prof. C. Wang

Research Institute of Materials Science

South China University of Technology

Guangzhou, 510640, China

Prof. Z. Zheng
Research Institute for Intelligent Wearable Systems
The Hong Kong Polytechnic University
Hong Kong SAR, China

Prof. Z. Zheng
Research Institute for Smart Energy
The Hong Kong Polytechnic University
Hong Kong SAR, China

Prof. J. Chang
Dongguan Key Laboratory of Interdisciplinary Science for Advanced Materials and Large-Scale Scientific Facilities
School of Physical Sciences, Great Bay University
Dongguan, Guangdong, 523000, China
Email: [changj@gbu.edu.cn](mailto:changj@gbu.edu.cn)


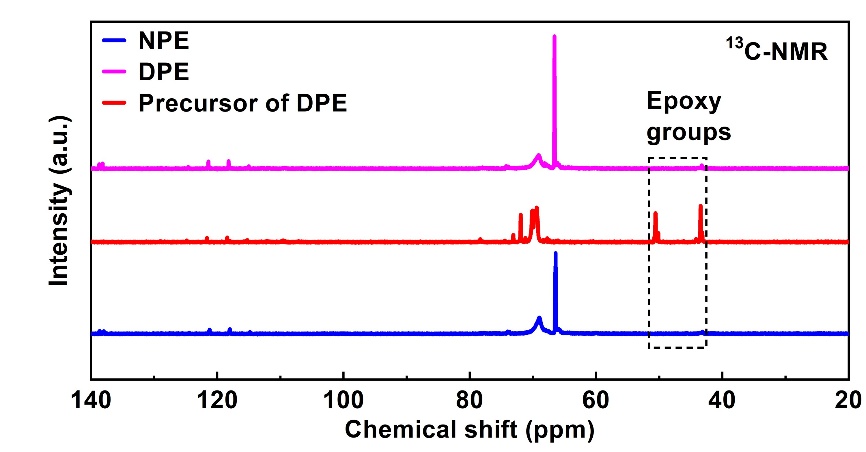


**Figure S1.** ^13^C-NMR spectra of NPE, DPE and its precursor.


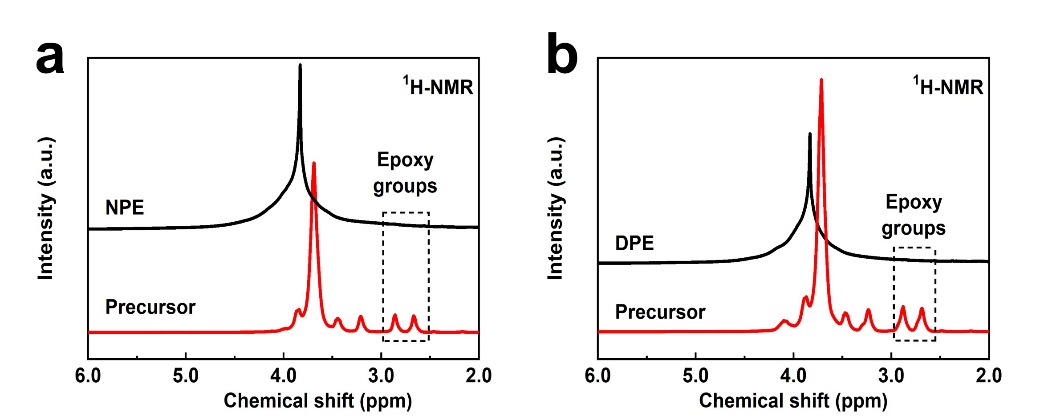


**Figure S2.** ^1^H-NMR spectra of (a) NPE and (b) DPE before and after polymerization.


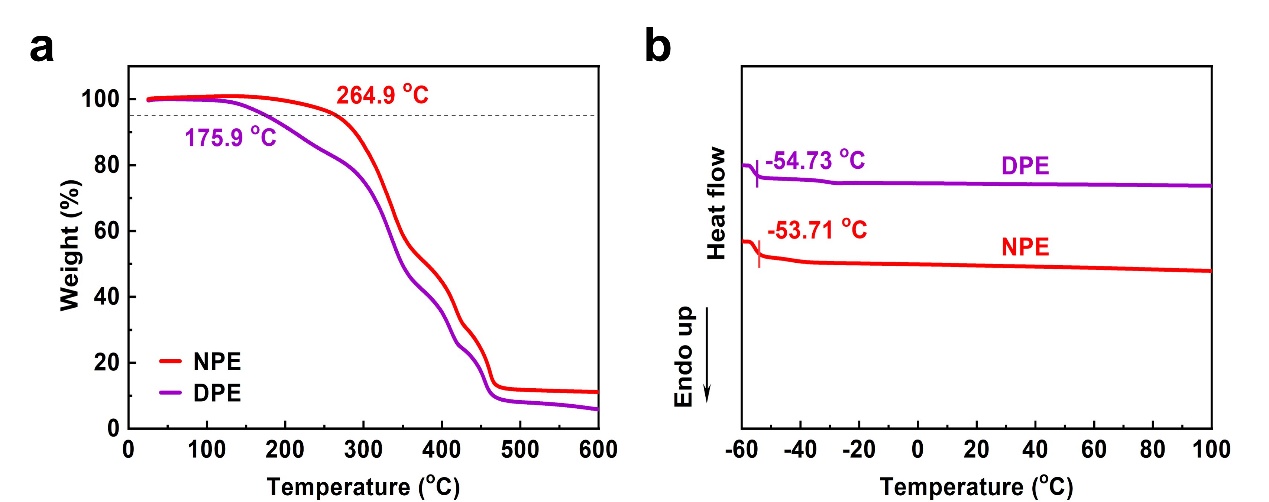


Figure S3. The thermal characterizations of NPE and DPE. (a) TGA and (b) DSC curves.


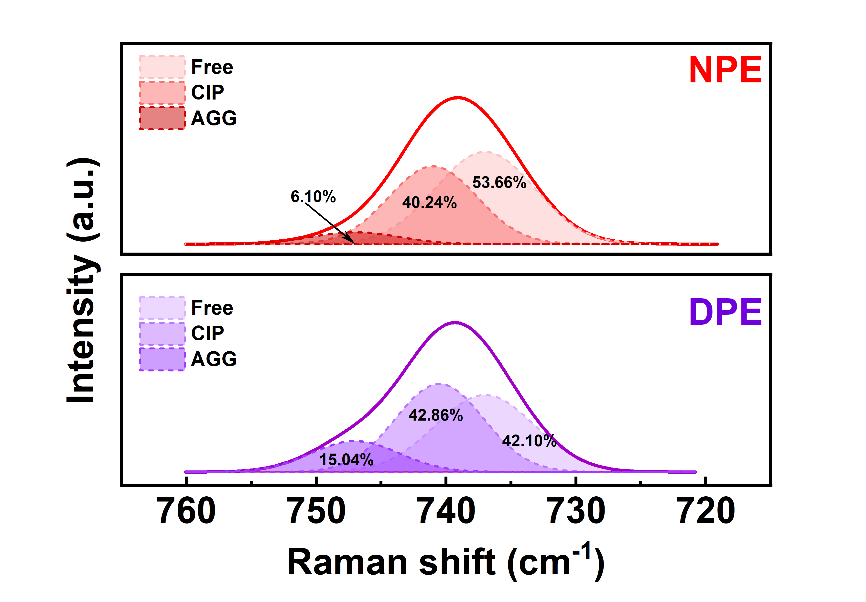


Figure S4. Raman spectra of NPE and DPE.


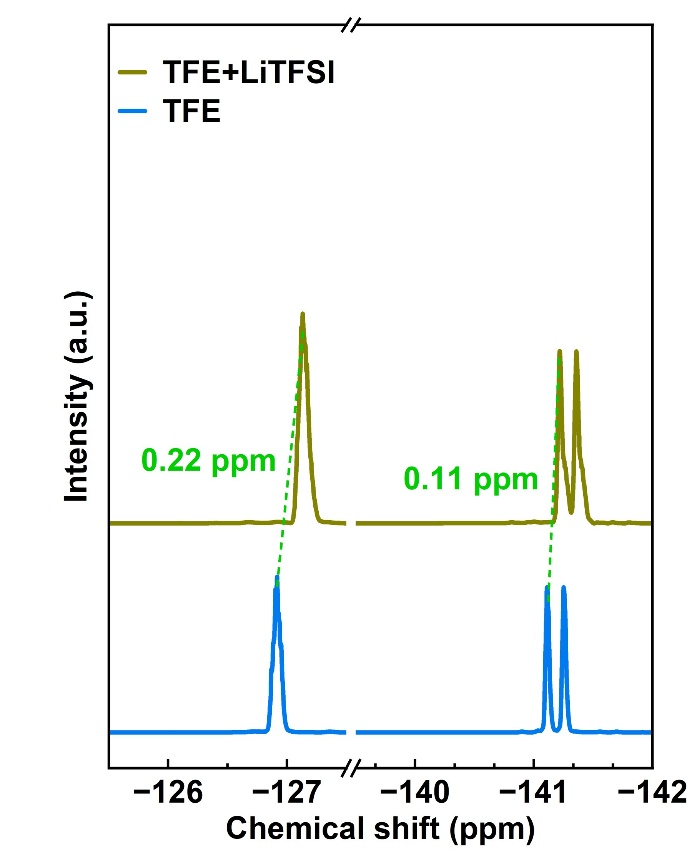


**Figure S5.** ^19^F-NMR for TFE with/without LiTFSI.


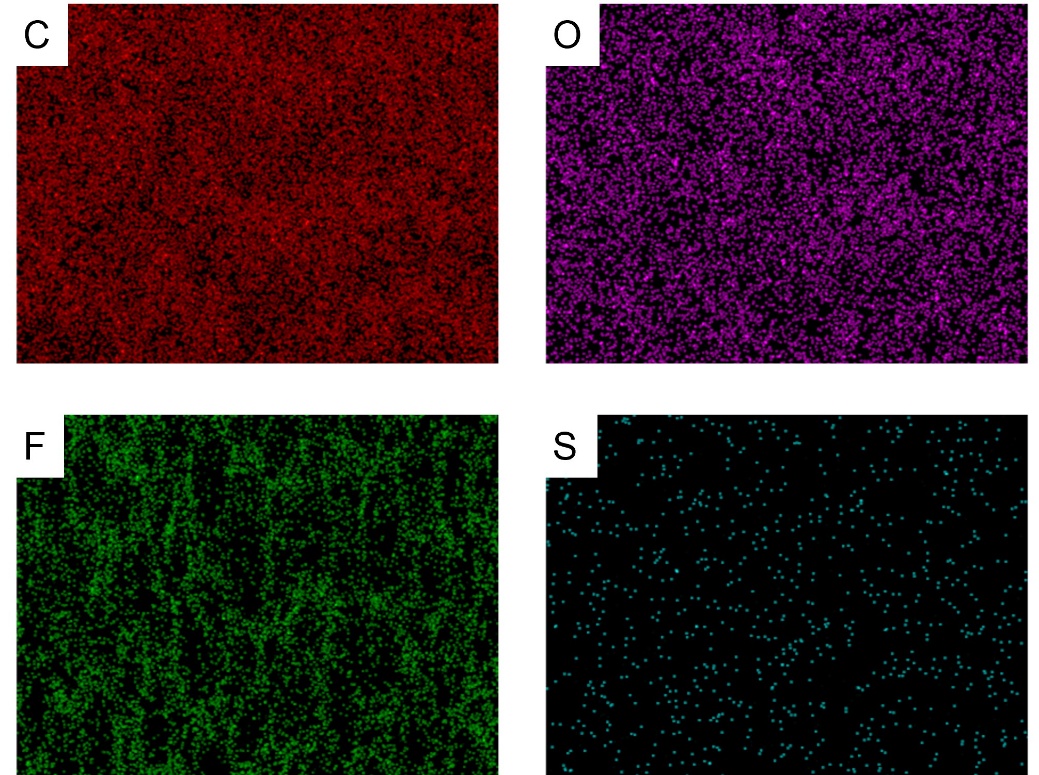


**Figure S6.** EDS mapping images of C, O, F and S in DPE.


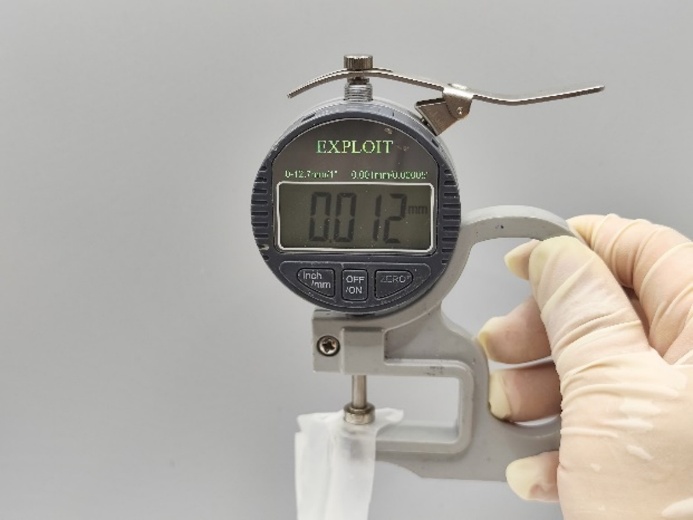


**Figure S7.** Digital photograph of the thickness for the composite film.


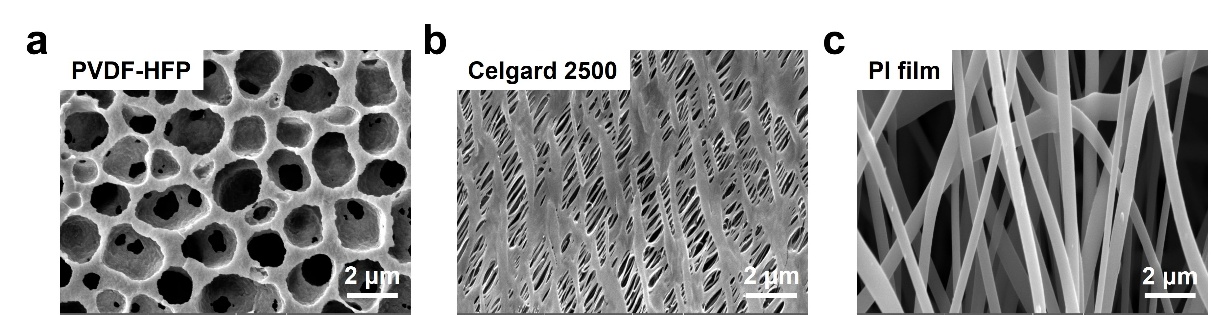


**Figure S8.** SEM images of (a) prepared mesoporous PVDF-HFP film, (b) commercial Celgard 2500 and (c) commercial PI film.


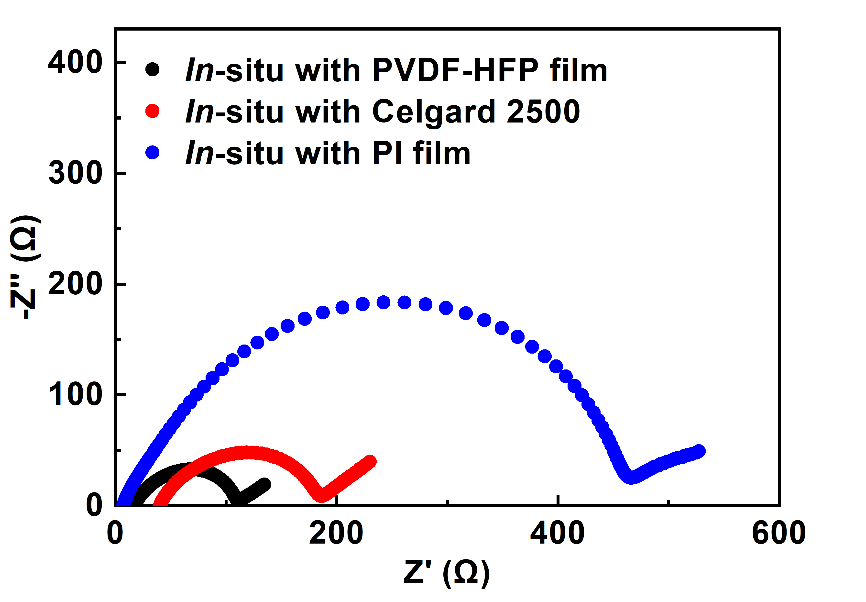


**Figure S9.** Nyquist plots of symmetric Li||Li cells with PVDF-HFP film, Celgard 2500 and PI film, respectively.


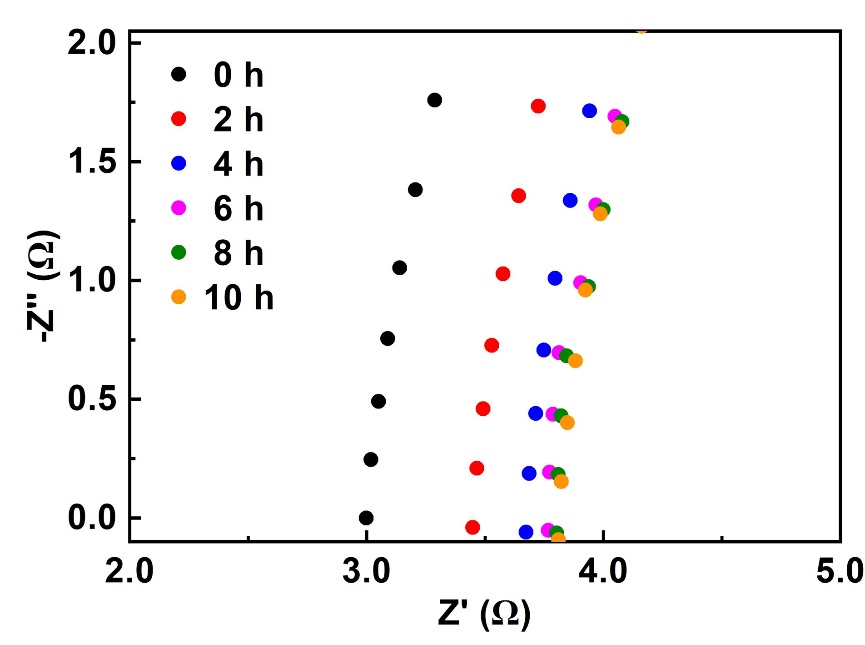


**Figure S10.** Nyquist plots of the SS||SS cell paring with composite film after heating for different hours.


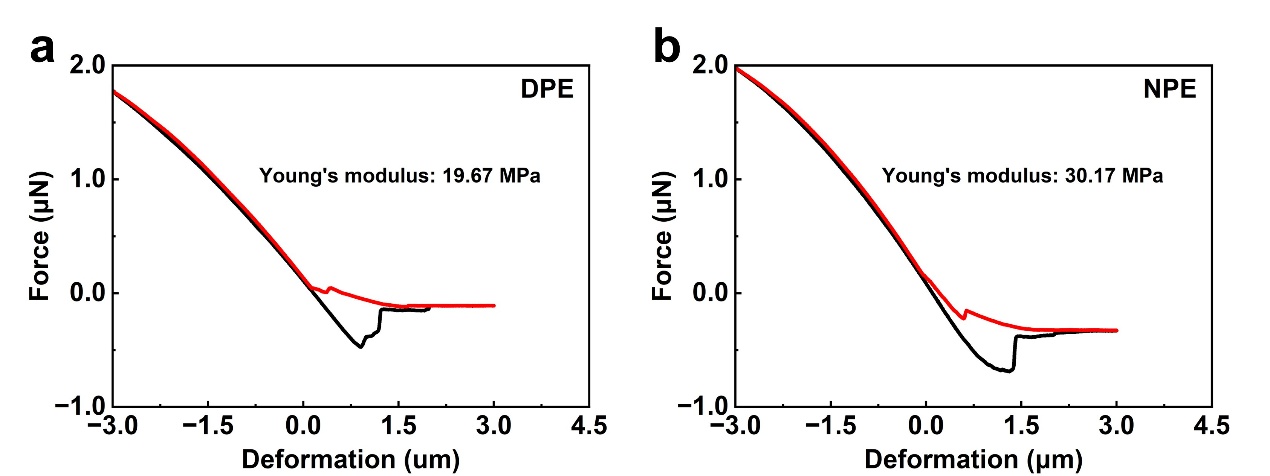


**Figure S11.** AFM force spectroscopy of (a) DPE and (b) NPE.


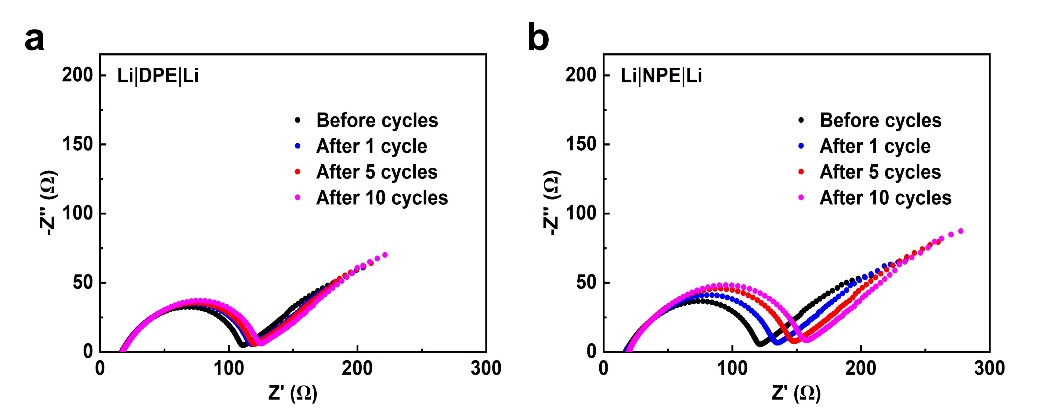


**Figure S12.** Nyquist plots of symmetric Li||Li cells pairing with (a) DPE and (b) NPE after different cycles.


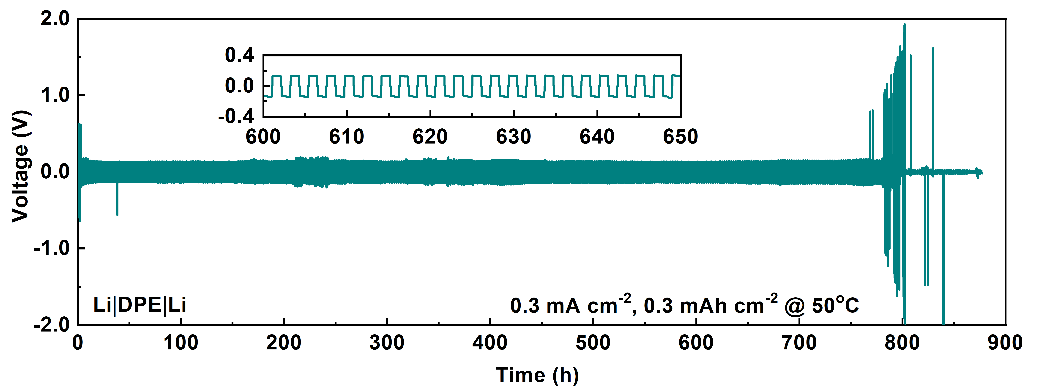


**Figure S13.** Cycling performance of Li|DPE|Li battery at 0.3 mA cm^-2^.


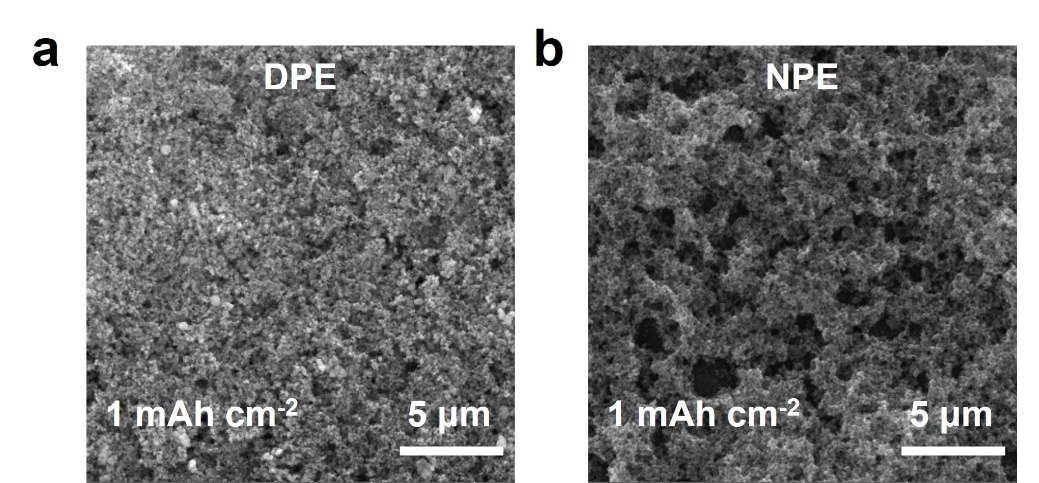


**Figure S14.** Morphologies of deposited lithium at the high capacity of 1.0 mAh cm^-2^ for Li||Li cells pairing with (a) DPE and (b) NPE.


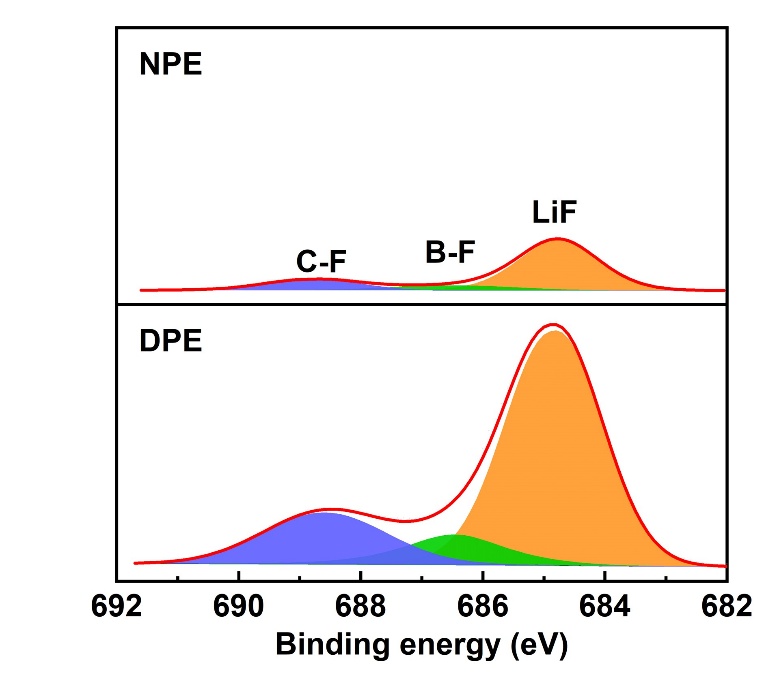


**Figure S15.** XPS spectra of NPE and DPE.


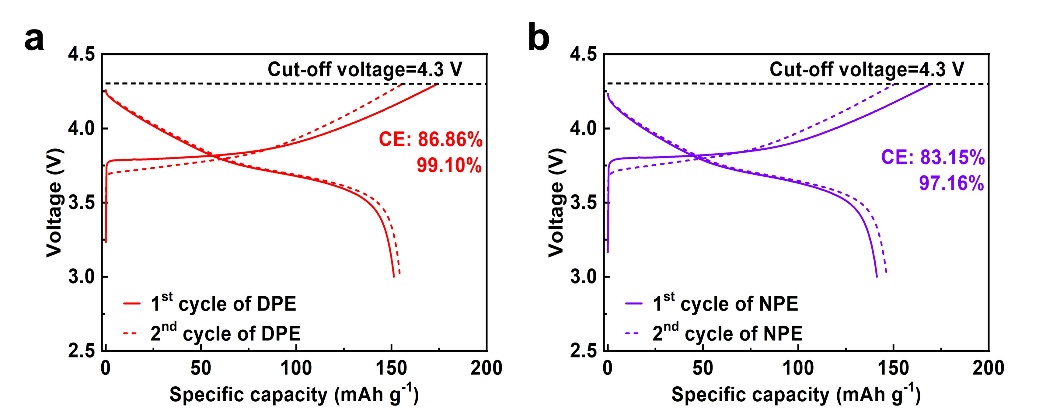


**Figure S16.** Charge/discharge profiles for the first two cycles of Li||NCM622 batteries pairing with (a) DPE and (b) NPE.


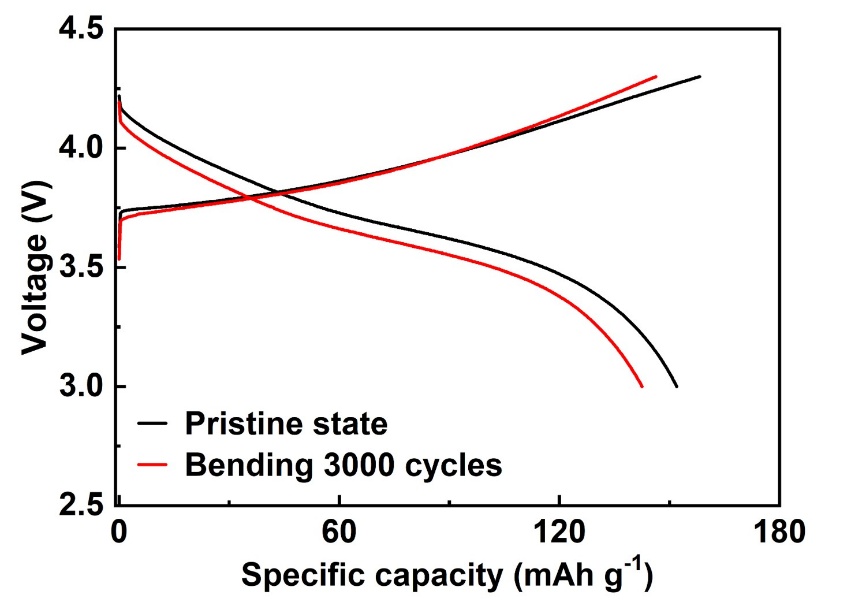


**Figure S17.** Charge/discharge profiles of assembled pouch cell before and after 3000 bending cycles.

**Table S1.** Surfacial element distribution of pristine and cycled NCM622 with NPE and DPE.

|  | C | O | F | S | Ni | Co | Mn |
| --- | --- | --- | --- | --- | --- | --- | --- |
| Pristine | 20.76 | 35.78 | 2.9 | 0.3 | 24.69 | 8.05 | 7.50 |
| Li/NPE/NCM622 | 30.95 | 34.85 | 3.79 | 0.58 | 18.38 | 6.01 | 5.43 |
| Li/DPE/NCM622 | 19.92 | 32.41 | 10.18 | 0.03 | 22.80 | 7.59 | 7.07 |
